# Diagnostic criteria to differentiate pathological procrastinators from common delayers: a re-analysis

**DOI:** 10.3389/fpsyg.2023.1147401

**Published:** 2023-07-06

**Authors:** Fred Rist, Margarita Engberding, Anna Hoecker, Johanne Wolf-Lettmann, Eva-Maria Fischbach

**Affiliations:** Clinical Psychology and Psychotherapy, Psychological Institute, University of Münster, Münster, Germany

**Keywords:** pathological procrastination, diagnostic criteria, treatment indication, latent class analysis, chronic procrastination, assessment

## Abstract

Detection and treatment of clinically relevant forms of procrastination would be greatly facilitated by diagnostic criteria as formulated for psychological disorders in the Diagnostic and Statistical Manual (DSM-5). In the present article, the steps for deriving and validating diagnostic criteria for pathological procrastination are described. In an online survey of a random sample of *N* = 10,000 German university students, 990 answered 13 items derived from the attempts in the literature to define procrastination, the Aitken Procrastination Inventory (API) and the Patient Health Questionnaire (PHQ-9). A subset of six items related to the first factor *onset delay* of the API was selected by Best Subset Multiple Regression (BSMR). A latent class analysis (LCA) of these six items sorted the students into six clusters. A cluster of *pathological procrastinators* (10%) was separated from the clusters of less impaired *habitual*, *average*, and *occasional delayers*. In addition, a cluster of *unconcerned delayers* (10%), with strong procrastination tendencies but little personal disadvantages, and a small cluster of *fast performers* (2%) emerged. The *pathological procrastinators* differed from all other clusters significantly on nine of the 13 items. They were older, had studied longer but had fulfilled less of their study obligations and were more depressed. The answer options of the six questions were collapsed into two categories (*procrastination feature present for at least half a year* or *absent*). These criteria were used for the clinical diagnosis of pathological procrastination. For a diagnosis, two fixed criteria (*delaying important tasks needlessly* and *strong interference with personal goals*) plus at least two of four additional criteria (*time spent procrastinating*, *time pressure*, *physical and psychological complaints*, *below performance potential*) must be met. This diagnostic rule captured 92% from the cluster of *pathological procrastinators* and 10% of the *habitual delayers*, but no one from the remaining clusters. Using these diagnostic criteria for clinical diagnosis and intervention decisions will facilitate the comparison and integration of the results from future studies of procrastination.

## 1. Introduction

### 1.1. Differentiating between common delay and pathological procrastination

#### 1.1.1. Missing diagnostic criteria

Procrastination, in the sense of delaying personally relevant actions to such an extent that the life quality of the person affected is impaired, has been discussed primarily as a problem of high school and university students. It is also common among the general population, according to epidemiological studies (Harriott and Ferrari, 1996; Steel and Ferrari, 2013; Beutel et al., 2016). However, when using the term “procrastination” with regard to the behavior of delaying the fulfillment of tasks or the implementation of plans, it is unclear whether one is referring to “pathological delay or procrastination” with severe consequences for one’s personal life, or to “common everyday procrastination” which the majority of people knows and has to cope with. Schouwenburg et al. (2004) point out that it has become the general opinion that procrastination represents an approximately normally distributed characteristic of a “normal behavior.” They call for a clear distinction between unplanned dilatory behavior and habitual or chronic delaying to be considered as trait procrastination.

Some authors suggest reserving the term “procrastination” explicitly for severe dysfunctional, irrational and detrimental delay of intended actions in contrast to everyday less pronounced forms and strategic variants of delay that are reasonable (e.g., Steel, 2007; Klingsieck, 2013). Svartdal and Løkke (2022) point out the difficulties of such a distinction in the absence of differentiating criteria and notice that the mentioned variants appear mixed in self-report scales at the expense of their validity.

Procrastination is not an acknowledged psychological disorder within the framework of the current scientifically approved diagnostic systems ICD or DSM. If one wishes to differentiate particularly serious extents of a dilatory behavior from less severe variants, criteria need to be defined to determine clinically relevant behavior that requires treatment (Saddler and Sacks, 1993). In view of this desideratum Milgram [Foreword in Ferrari et al. (1995), p. xi] already made the “modest proposal: To constitute a national task force to develop criteria for diagnosis, assessment, and treatment of procrastination disorders [.] the same kind of operationally defined criteria found in the Diagnostic and Statistical Manual (DSM; American Psychiatric Association, 2013) for established psychiatric and behavioral disorders.” In the preface to the same influential book on procrastination (Ferrari et al., 1995), Ferrari too asked for diagnostic criteria as for other disorders depicted in the DSM.

Subsequently many studies emphasize the need for a consensus about the definition of dysfunctional procrastination for research and practice, but strangely enough no attempt has ever been made to establish and empirically substantiate such diagnostic criteria for procrastination.

#### 1.1.2. Differentiating subgroups which vary in procrastination behavior

Diagnostic rules can be established with more confidence if the information on which a diagnosis is based sorts subjects into clusters which correspond to the diagnostic rules. The task of establishing diagnostic criteria for procrastination will be simplified if specific information on procrastination behavior can be collected which leads to such a “natural” ordering of persons.

Grunschel et al. (2013) and Rozental et al. (2015) summarize studies with cluster or factor analytic methods applied to differentiate varieties of procrastination behaviors or groups of people who procrastinate. Rozental et al. (2015) examined Swedish students who had responded to an offer of internet-based cognitive behavior therapy for procrastination. Three procrastination scales and the Susceptibility to Temptation Scale were used to determine levels of procrastination. Depression, anxiety, and quality of life were also assessed. In a five-cluster-solution a *severe procrastinators* cluster (22%) scoring highest on all procrastination scales and three more clusters with lower procrastination severity were found. In addition, a depressive cluster was found, which may be due to the therapeutic offer of the study. Grunschel et al. (2013) asked for reasons of academic delay, recorded by 14 sets of items assessing working habits. They found procrastination in the clusters of *worried/anxious* and of *discontent with studies* clusters. They also obtained a cluster of *pressure seeking* not emotionally affected by their procrastination. With questionnaires assessing impulsivity, emotion regulation, self-esteem and global motivation, Rebetez et al. (2015) found four clusters with different scores on the Pure Procrastination Scale (PPS; Steel, 2010). High PPS scores were reported for the *emotional* and the *unregulated* cluster.

According to these studies subjects may be sorted into groups with different varieties and degrees of procrastination behavior. These results have not yet been applied to delineate diagnostic criteria to detect and differentiate seriously self-damaging from less severe forms of procrastination behavior. Also, these studies used sum scores of questionnaire answers, while diagnostic criteria in general are based on features which are recognized as present or absent depending on their frequency, duration, and extent. Alternatively, clusters may be formed based on single ordinal items which can be translated into dichotomous criteria if they differentiate between pathological procrastination and less self-handicapping variants.

#### 1.1.3. Benefits of a categorical diagnosis

Establishing criteria for a diagnosis based on psychological facets of a person’ss life is difficult if none of the information regarding the problematic psychological areas can be obtained in the form of signs and symptoms. Most symptoms dealt with in Psychiatry and Clinical Psychology are continuous and are designated as criteria for a disorder when they surpass defined boundaries.

A valid and widely accepted categorical case definition is a prerequisite to investigate dysfunctional procrastination as a psychological disorder. A categorical case definition is needed for differential diagnosis of various patient groups as a basis for research on prevalence, on specific manifestations, on causes and practical decisions about treatment indications. If an agreed classification is lacking, epidemiological and psychological studies have to be limited to correlative findings (cf. Klingsieck, 2013; Steel and Ferrari, 2013; Beutel et al., 2016).

For several years we follow the above recommendations and try to determine the degree of intense, pervasive and chronic delay that we suggest to designate as “pathological procrastination” (Frings, 2008; Wolf, 2011; Höcker et al., 2017). Instead of describing persons only dimensionally according to the extent of their procrastination, we aim for a clinical diagnosis of procrastination which follows the logic of the DSM and will assign people clearly to separate classes: pathological procrastinators (in need of behavioral change or treatment) versus common delayers and “non-delayers.” We expect that the introduction of such diagnostic criteria – similarly to the effects of introducing the diagnosis “Post-Traumatic Stress Disorder (PTSD)” in earlier versions of the DSM – will substantially enhance clinical research activities on procrastination.

#### 1.1.4. Diagnostic criteria in DSM-format

Based on clinical-psychological experiences with patients at our procrastination outpatient clinic and investigations of large samples we conceptualized diagnostic criteria analogous to those used to categorize and describe disorders in the DSM (American Psychiatric Association, 2013). Here, “A mental disorder is a syndrome characterized by clinically significant disturbance in an individual’s cognition, emotion regulation, or behavior that reflects a dysfunction in the psychological, biological, or developmental processes underlying mental functioning. Mental disorders are usually associated with significant distress in social, occupational, or other important activities” (American Psychiatric Association, 2013, p. xxxi). The criteria are organized as A-, B-, C-, and D- or more criteria. A defined part of them must be met with regard to the intensity, duration, and frequency of occurrence of the symptoms during lifetime and at present. The first “A”- criterion comprises the main signs and symptoms of the disorder in question that must exist to justify the diagnosis. The further criteria ask for features and additional information regarding possible variations of the symptoms or their impact on the individual’s life. Finally, the last criterion always requires differentiation from similar signs and symptoms that may be caused by other conditions and are not attributable to the disorder in question.

The identification of procrastination as clinically relevant disorder requires evidence of considerable psychological deficits and long-term problems concerning essential domains and tasks in the everyday life of affected persons.

In the next sections we will explain the initial development process: First we report essential characteristics of procrastination outlined in the relevant special literature used as basis for the construction of a questionnaire for our data collection. Then we describe the first attempt of deriving criteria, which resulted in the original PDC 6/8 (Höcker et al., 2017; see Appendix) and the reasons for the continuation of the data analysis in the present study. This is followed by the presentation and discussion of the current re-analysis and the resulting revised criteria solution PDC 4/6.

### 1.2. Procrastination characteristics as starting point for diagnostic criteria

#### 1.2.1. Lack of agreement on definition and differing prevalence estimates

The components of definitions and diagnostic characteristics of procrastination put forward in the past include extensive combinations of different features and degrees of procrastination. Thus, they do not capture the same procrastinating persons and consequently lead to differing prevalence estimates of which we will give some examples (In the overview below the Cursive letters in square brackets refer to the final list of procrastination facets providing the pool for the diagnostic questionnaire items of our study in the same order).

Solomon and Rothblum (1984) found that 50% of their student sample judged their own procrastination behavior to be a “moderate or severe problem.” According to Rothblum et al. (1986), 40% of the students in their study stated, “that they always or nearly always procrastinated on studying for exams and that such procrastination nearly always or always made them feel anxious” [*f*]. In a study by Day et al. (2000), 50% of the questioned American college students reported having problems in their studies [*l*] as a result of their postponement. Briody (1979) used a somewhat stricter definition of “problem procrastinators” including that “their tendency to put things off interfered with both their grades and their enjoyment of life” [*k,e*] and found a prevalence of 20% among students. Klassen et al. (2010) report higher prevalence estimates with 28% of students stating “procrastination negatively influencing their academic functioning” [*k*]. Steel (2007) reports widely differing prevalence estimates with a range from 15 to 95% (!). This discrepancy is certainly partly due to the different samples investigated, but mainly attributable to the different approaches to assess procrastination and to set cut points to determine its presence. The lack of agreement on a common definition may explain the widely differing prevalence estimates of research groups.

#### 1.2.2. Self-regulation deficit and relevant task-avoidance

Many definitions of procrastination emphasize the discrepancy between the intended action and the actual behavior as a core feature (Lay and Schouwenburg, 1993; Ferrari et al., 1995; van Eerde, 2000; Steel et al., 2001; Steel, 2007, 2010) [*a*]. Solomon and Rothblum (1984; p. 503) mention the aspect of “needlessly delaying tasks to the point of experiencing discomfort” [*a,e*]. According to Silver and Sabini (1981), the preference for less important activities is an integral part of procrastination: “Someone meets a less important obligation in order to procrastinate a more important obligation” [*c*].

It is repeatedly stated that the postponed task is often experienced as aversive. Thus, according to Milgram (1988), tasks that are perceived as unpleasant are postponed. Blunt and Pychyl (2000, p. 165) take a similar view, explaining postponement as “when individuals perceive their projects as being aversive in that these projects are boring, frustrating, done resentfully, forced upon them by others and are generally more stressful, less meaningful and less structured” [*d*].

Some definitions, e.g., Ferrari et al. (1995; p. 3f) emphasize these motivational characteristics of procrastination as “task avoidant behavior” and state an inability to meet deadlines and “a frustrating and ultimately self-defeating pattern” [*d*,*i,l*]. They cite earlier explanations of procrastination including several components, such as Silver (1974; p. 6), who described it as putting the task off “past the optimal time it should be initiated to guarantee the maximal likelihood of successful completion” [*b*,*j,k*] or Milgram (1991; p. 11), who combined four characteristics: postponement [*a,b*], concerning important tasks [*a*], substandard behavioral product *[i,k]*, and resulting in a state of emotional upset [*e,f*].

van Eerde (2003) considers the failure in self-regulation (see also Ferrari, 2001; Shalev, 2018) in the sense of a preference for a short-term, immediate reward compared to a longer-term one as deficit in delaying gratification and a lack of impulse control leading to the experience of negative affect and guilt [*e*]. Accordingly, Sirois and Pychyl (2016) refer to procrastination as a strategy for emotion regulation providing short-term mood-repair by eliminating current uncomfortable feelings at the expense of future well-being. Wypych et al. (2018) and Mohammadi Bytamar et al. (2020) also found connections between procrastination and difficulties in emotion regulation especially in coping with unpleasant emotions.

Steel’s very influential characterization of procrastination as “voluntarily delay an intended course of action despite expecting to be worse off from the delay” accepting even negative consequences like the resulting risk of poorer performance underlines the irrational ingredients [*a,b,k*] (Steel, 2007; p 66f. See also Pychyl and Sirois, 2016). The phrase “intended course of action” permits the implication that procrastination - within a comprehensive motivational and volitional model of intent realization such as the Rubicon Model – must not only refer to the core point of action initiation, but also to other phases and phase transitions of the process: Thus decisions for the pursuit of certain goals, the planning of concrete steps or the consistent and purposeful continuation of already begun actions despite difficulties can also be postponed (cf. Heckhausen and Gollwitzer, 1987; Svartdal et al., 2020).

#### 1.2.3. Keeping the intention in mind

According to the experience of clinicians and counselors, chronic procrastinators while devoting themselves for an extended period to alternative activities often remain in a permanent mental preoccupation with the deferred activity. This occurs despite strong avoidance tendencies and attempts to suppress such thoughts and connected worries [b,g,h]. Many participants of our Anti-Procrastination-Trainings^1^ stated that they generally suffered from not being able “to switch off” and constantly were thinking about what “should actually be done.” They realize that they are procrastinating and wasting precious time, although in the process they can’t really enjoy the substitute activity. The separation between effective working time and relaxing leisure time is becoming increasingly blurred and both parts are impaired and spoiled.

#### 1.2.4. Impairment of mental and physical well-being

Schraw et al. (2007) found that stress caused by procrastination has a negative effect on health and mental well-being. Rothblum et al. (1986) stated already earlier that procrastinators report physical symptoms more often than their not delaying fellow students. Tice and Baumeister (1997) found that procrastinators, especially toward the end of the semester, generally experience more stress with approaching deadlines and exams, go to the doctor more frequently and report more symptoms of illness [*f,j*]. In their study with a large representative German sample Beutel et al. (2016) found that procrastination was associated with higher stress and anxiety and with reduced life satisfaction in various domains. The risks of procrastination for mental and physical health and for well-being are reflected in detail in various articles in the anthology edited by Sirois and Pychyl (2016) *[f,j]*. They report empirical findings of significant associations between procrastination and lower positive affectivity or higher negative affectivity and a relation to overall worse emotional well-being. The connection of procrastination and depression will be considered below.

#### 1.2.5. Time pressure and losses in goal achievement

Lay (1986) defines procrastination as the tendency to postpone actions necessary for the achievement of personal goals. He focuses primarily on the outcomes of procrastination, namely pressure of time or not finishing tasks at all, and disadvantages for personally relevant goals [*i,j,l*]. Steel (2007) describes procrastination as the conscious choice of an action that does not help to approach one’s own goals. Frequent and regular postponement often leads to enormous time pressure for the punctual completion of certain tasks or, in the worst case, even to missing deadlines for their completion [*i,j,l*] (Beswick et al., 1988; Ferrari et al., 1995; Senecal et al., 1997; Steel, 2007). The resulting achievement impairment is a commonly reported consequence of procrastination, mainly due to the lack of time left for the execution of important extensive tasks [*b*,*k*] (Ferrari, 1994; Steel, 2007; Kim and Seo, 2015). In this context procrastination preventing a realistic feedback about the performance can even be considered as active self-handicapping strategy in a beneficial self-esteem-maintaining function (Ferrari, 1991; Beck et al., 2001; Strunk and Steele, 2011). Rothblum et al. (1986) report significant negative correlations between procrastination and the grade point average and assume that procrastination generally leads to poorer academic performance [*k*]. Steel et al. (2001; p. 95) claim that “procrastination is an excellent predictor of performance.”

#### 1.2.6. Failure, remorse, and depression

The strong connection between procrastination and depressed mood has been confirmed in many studies (Steel, 2007; Beutel et al., 2016; Rozental et al., 2022) and seems psychologically very plausible: The repetitive experience of discrepancy between aspired goals and actual performance deficits, between personal intent and non-compliance, in combination with resulting sensations of disappointment, remorse, guilt, and shame significantly dampens mood and self-esteem of affected persons. This may lead to depressive symptoms, which in turn - like a vicious circle - lead to increased anticipation of failure, reduced drive and thus also to aggravation of the procrastination tendency. In a study about the effects of shame and guilt on procrastination Oflazian and Borders (2022) found that depressive rumination in form of brooding and reflective pondering mediates the relationship in the sense of increasing procrastination [*b,e,f]*.

#### 1.2.7. Interpersonal problems

Procrastinators often have the image of being lazy, listless, or unreliable (van Eerde, 2003; Blunt and Pychyl, 2005). Their postponing behaviors cause incomprehension and can lead to interpersonal problems and conflicts with family members, partners and friends (Fydrich, 2009; Rozental et al., 2014). Beutel et al. (2016) report a connection between procrastination and lack of a partnership. Studies on the consequences of workplace procrastination find not only professional disadvantages for chronic procrastinators, but also problems in interpersonal relationships with colleagues and superiors (Skowronski and Mirowska, 2013; Abbasi and Alghamdi, 2015) [*m*].

#### 1.2.8. Chronic procrastination as dysfunctional complex of various components

Abbasi and Alghamdi (2015) conceptualize “chronic procrastination” as a self-regulatory failure in form of “a deliberate and repetitive postponement of either starting or finishing a task such that the delay leads to subjective discomfort” and use this term for a broad conglomerate of abnormalities. Referring to Ferrari (2001), Burka and Yuen (2007), Ferrari (2010), Ferrari and Diaz-Morales (2014), they specify, among other phenomena, higher levels of stress and anxiety, negative consequences for a healthy lifestyle, deficits in task and time management skills, lack of impulse control and work discipline and lower performance and accuracy as compared to non-procrastinators.

Klingsieck (2013; p. 25f) gives an overview of various definitions intending “to provide a clear distinction between procrastination and functional forms of delay”: She offers a conceptual combination of seven constituent aspects concerning the quality of delayed acts (covert or overt, start or completion intended, necessary or of personal importance) and the characteristics of the delay (voluntary, unnecessary, or irrational, despite negative consequences, connected with subjective discomfort). She argues that dysfunctional delay and functional strategic delay share the first four components and that they differ especially with regard of the three last characteristics.

### 1.3. Summarizing definition components for deriving criteria

#### 1.3.1. Repeating features

Despite the differences in the numerous definitions of procrastination and its consequences, certain aspects are frequently repeated across diverse descriptions. From this presentation of various facets of procrastination, we selected those which were repeatedly mentioned in the literature as typical features of aggravating forms of unintended and serious forms of delay. They served as starting points for the choice of essential characteristics and the formulation of suitable items necessary to achieve a diagnosis of procrastination.

To arrive at valid and reliable diagnostic criteria we collected essential characteristics in accordance with the different attempts of definition mentioned above. Reviewing the literature, we specified 13 determinants of clinical relevance that reappear in various definitions of procrastination including reference points, behavioral aspects, motives, and consequences:

[*a*] Delaying important tasks needlessly *(PDCQ Item 1)*
[*b*] Time spent procrastinating *(PDCQ Item 3)*
[*c*] Preferring less important activities *(PDCQ Item 7)*
[*d*] Task aversion *(PDCQ Item 8)*
[*e*] Discomfort/Remorse *(PDCQ Item 10)*
[*f*] Physical or psychological complaints *(PDCQ Item 5)*
[*g*] Keep thinking about task *(PDCQ Item 11)*
[*h*] Suppressing thoughts *(PDCQ Item 12)*
[*i*] Tasks not completed *(PDCQ Item 4)*
[*j*] Time pressure *(PDCQ Item 4)*
[*k*] Below performance potential *(PDCQ Item 6)*
[*l*] Interference with personal goals *(PDCQ Item 2)*
[*m*] Relationships affected *(PDCQ Item 9)*

The list above contains already the item numbers of the resulting final Procrastination Diagnostic Criteria Questionnaire (PDCQ-12) in round brackets (for combining [i] and [j] in Item 4 see Appendix).

#### 1.3.2. Derivation of initial diagnostic criteria for procrastination (PDC-5/8)

These 13 facets served as guidelines to formulate 13 questions with a Likert type answer format (see Table 1) to assess the core phenomena of procrastination to be used for the final set of diagnostic criteria (Frings, 2008; Wolf, 2011). In a cross-sectional study the 13 questions were administered together with the procrastination questionnaire API (Aitken, 1982) and further questionnaires to 990 students (see the section “2. Materials and methods” for details). By use of BSMR models those items that identified procrastinators most precisely as determined by their API value were selected. The mean score for the first factor *onset delay* (*M* = 2.89, SD = 0.50) of *N* = 350 successive clients who had asked for treatment in the specialized procrastination outpatient clinic at the University of Münster from 2004 to 2010 because of their problems with procrastination was used as threshold for differentiation between procrastinators and non-procrastinators (Höcker et al., 2017) both in examining the first proposal and the here reported revised version of the criteria (see below). The first resulting set contained a combination of eight diagnostic criteria. Two of these were necessary ones and three from the remaining six criteria had to be met (PDC-5/8, see Appendix). Within the initial eight criteria two substantive groups each with four items can be distinguished: The first group of items assesses characteristics of procrastination behavior (delaying important tasks needlessly, time spent procrastinating, preferring less important activities, task aversion). The second group assesses negative consequences (time pressure, below performance potential, interference with personal goals, physical and psychological complaints).

**TABLE 1:** Procrastination questions, answer format, answer frequencies and correlations with API and PHQ-9.

Item	Questions verbatim	Questions abbreviated	Answer format	Choice of response options (%)^a^					Criterion reached (%)	r_*API*_^b^	r_PHQ–9_^c^
1	To which extent did procrastination hinder you from reaching your personal goals?	Interference with personal goals	1) Not at all 5) Very strongly	24	35	24	12	5	B 17	0.57	0.41
2	Have you delayed very important tasks past the adequate point in time, despite sufficient time for it had been available?	Delaying important tasks needlessly	1) Not at all 4) Almost every day	5	56	29	10		A 39	0.62	0.32
3	Remember important tasks you procrastinated: How much of the time available for the task did you spend procrastinating?	Time spent procrastinating	1) None 5) More than 75%	2	37	33	21	6	C1 27	0.63	0.33
4	Remember important tasks which you wanted to finish during the past half year. How many of these did you complete only under great time pressure because of procrastination?	Time pressure	1) None 5) More than 75%	18	34	21	18	10	C2 28	0.60	0.27
5	Physiological and psychological complaints	Complaints	More than 5 complaints						C3 26	0.36	0.65
6	Starting from a performance potential of 100%: To which extent (%) did you remain below your performance potential because of procrastination?	Below performance potential	1) No loss 5) More than 75%	41	39	15	6	0	C4 21	0.50	0.37
7	Did you choose other, less important activities, despite you wanted to start with your more important task?	Preferring less important activities	1) Not at all 4) Almost every day	3	60	29	8			0.54	0.30
8	Remember important tasks which you wanted to finish during the past half year. How many of these plans did you complete not at all because of procrastination?	Tasks not completed	1) 0% 5) More than 75%	46	37	11	5	2		0.47	0.32
9	Did the tasks you postponed cause reluctance or aversion?	Task aversion	1) Not at all 4) Almost every day	13	54	22	11			0.39	0.45
10	Has procrastination affected your personally important relationships to significant others?	Relationships affected	1) Not at all 4) Almost every day	53	39	6	2			0.36	0.41
11	Did procrastination cause strong discomfort or remorse?	Discomfort or remorse	1) Not at all 4) Almost every day	7	37	28	29			0.36	0.45
12	Do you try to suppress thoughts about the postponed task?	Suppressing thoughts	1) Not at all 4) Almost every day	37	44	13	6			0.27	0.32
13	Do you keep thinking about the postponed task while procrastinating?	Keep thinking about tasks	1) Not at all 4) Almost the whole day	3	25	31	41			0.16	0.31

*n* = 990.

^a^Underlined response options yield dichotomous criteria for the diagnostic rule.

^b^r_API_: correlation of Aitken procrastination inventory factor 1 sum scores with procrastination items.

^c^r_PHQ–9_: correlation of patient health questionnaire for depression sum score with procrastination items.

The relevant criteria items selected by Best Subset Regression Analysis providing a variance explanation of 67% were checked for the optimal combination of specificity and sensitivity values using ROC analyses. From a clinical perspective, strict guidelines for the assignment to a disorder requiring treatment are preferred. The predictive quality with a very good specificity of 0.95 and a worse but still acceptable sensitivity of 0.54 was judged as preliminarily sufficient. Nevertheless, applying the PCD-5/8 a relatively high prevalence of 11.6% was found for the study sample (Wolf, 2011).

#### 1.3.3. Previous use of the initial criteria PDC 5/8

After its development the corresponding PDCQ-8 became an integral part of a test battery in an anonymous online self-test to screen for procrastination and connected aspects and disorders such as Depression and ADHD accessible for all population groups. It has meanwhile collected data from participants in the six-digit range. For example, in a study with 16,614 completers of this self-test during 16 months a procrastination prevalence rate of 33% (!) was found, certainly due to the strong self-selection of those interested in the self-test (Engberding et al., 2017). In addition, for 1 year the PDCQ-8 was included in the standard diagnostics for all patients in the general psychotherapy outpatient clinic of the psychological department. This allowed to collect data for a comparative prevalence estimate for a “natural” clinical sample of *N* = 269 patients. Here the proportion of distinctive, treatment requiring procrastinators at 25% was lower than in the sample of the online-test but significantly higher than in the original cross-sectional sample which is the focus of the here presented re-analysis study.

In the meantime, the PDCQ-8 together with diagnostic instruments for procrastination is routinely filled out by patients in the general university psychotherapy outpatient clinic. It will be presented to all clients who answer three screening questions about procrastination affirmatively in an initial questionnaire. If patients then meet diagnostic criteria their therapists are offered supervision to use specific diagnostic instruments and interventions.

Rozental et al. (2022) had applied this diagnostic rule to a sample loaded with seriously procrastinating participants. This sample was divided at the median of the scores of the Pure Procrastination Scale (PPS; Steel, 2010) into severe cases “in need of support” and less severe cases. Below the median 17% and above the median 74% met the PDC-5/8.

### 1.4. The current study

#### 1.4.1. Critical appraisal of the initial criteria

The initial PDC-5/8 version is based on a rather liberal selection of criteria. Although the predictive contribution of *below performance potential* and *preferring less important activities* to the correct classification as procrastinator or non-procrastinator was only small, they were included in the catalog of criteria mainly due to comprehensive diagnostic interest and for content reasons because of their frequent occurrence in definitions. The same holds for the inclusion of the poorly selective item on task aversion. A more restrictive and tighter solution would be preferable.

In addition, the low sensitivity of the PDC-5/8 is unsatisfactory. The API values for *onset delay* chosen to guide the selection of diagnostic items in the BSMR might not necessarily reflect the complex “reality” of clinically relevant procrastination. However, it was at the time one of the best reference points in particular since the corresponding data of many clients seeking advice for their chronic and detrimental delay were available.

#### 1.4.2. Purpose and steps of the re-analysis

Our previous proposal of diagnostic criteria (PDC-5/8, see Appendix) comprised eight of the 13 procrastination items. The derived diagnostic rule of five criteria to be met from these eight has been described above, and in Höcker et al. (2022) and in Rozental et al. (2022). A re-analysis of the data shall focus on a stricter derivation of reduced criteria and their verification in a cluster analysis. A stricter item selection in the course of the BSMR is intended to reduce the number of criteria to highly predictive ones. This also promises an increase in stability for the desired reliable capture of the seriously affected persons. Considering various subgroup configurations might allow us to more precisely identify pathological procrastinators in contrast to other less problematic groups.

Since this re-analysis relates to the same data from which the previous version of criteria was derived a large part of the methods and materials were used both in the initial phase of the study and in the revision phase that is in the focus here. To impart understanding for the reason and for the starting point of the renewed evaluation presented here the previous methodical procedure had to be sketched already superficially in advance. In the next section “2. Materials and methods” used for the entire data collection and evaluation and for the additional procedures are explained in more detail. In the following sections the steps and results of the criteria revision are described and discussed.

## 2. Materials and methods

### 2.1. Participants

A random sample of 10,000 students from the University of Muenster was invited via E-mail to participate anonymously in an online survey concerning their personal experience with procrastination that would take about 25 min. As an incentive for participation, book vouchers were raffled. From all 1 833 respondents, 1,597 started to fill out and 1,041 (10.4%) completed the questionnaire. Fifty-one participants had to be excluded because of excessive time demands or incongruous answers. The data of *n* = 990 participants who had answered all questions (65% female and 35% male) were analyzed. Their mean age was 23.82 years (SD = 3.4). They had attended on average 6.2 semesters (SD = 4.6). Their fields of study covered the entire spectrum of studies offered at the university. Overall, 73.5% of the study participants stated to have met all requirements for their studies within the standard time.

### 2.2. Procedure

The questionnaire presentation was controlled by the *unipark* survey software,^2^ which provided a secure online interface. The questionnaire was accessible for ten days. The participants were informed about the research aims and asked to answer every item and to submit their informed consent. The software did not proceed to the next item if an item had been skipped. The instruction to the questionnaire contained an exposition of the data security measures taken and the rights of the participants to end the questioning at any time. The questionnaires started with demographic and study information followed by the procrastination questions prepared to provide diagnostic criteria. In addition, an established procrastination questionnaire and a depression screening questionnaire were presented.

### 2.3. Instruments

#### 2.3.1. Procrastination items

Identification of procrastination as clinically relevant disorder requires evidence of considerable psychological deficits and long-term problems concerning essential domains and tasks in everyday life. Diagnostic rules to decide on the presence of a psychological disorder rarely rely on qualitative attributes which are present or absent. In general, symptoms crucial for the diagnosis of a disorder must have passed a threshold of frequency, duration, or intensity to justify a certain diagnosis. The suggested diagnostic criteria for pathological procrastination follow this tradition.

To arrive at the intended diagnostic criteria, thirteen procrastination questions were formulated which capture essential features of procrastination named in the literature. To ensure that the procrastination symptoms do not only exist fleetingly but are stable during a longer period, the half-year criterion for the minimum duration was emphasized in the instruction for the questionnaire. A criterion of duration of at least half a year is demanded in the DSM for various mental disorders. Table 1 displays the wording of the 13 questions together with abbreviations which we will use in the text and in the other tables. The items are arranged to present first those six items which were selected for the diagnostic criteria, followed by the remaining seven items in descending order according to their correlation with the API first factor *onset delay*. The complete final questionnaire with all response options is presented in the Appendix.

The procrastination questions asked for frequency, intensity, or extent of the diverse aspects of procrastination behavior in a Likert scale format with four or five response options. Frequency items were to be answered by choosing one of four response options from *not at all* up to *almost every day*. Intensity items offered five response options from *not at all* up to *very strongly*. Items asking for a quantitative effect of a procrastination behavior offered five response options asking for a percentage value from *does not apply* to *more than 75%* in 25% steps. To assess the clinical impact, the questionnaire asked for the presence of four physical and five psychological complaints. Physical complaints were muscle aches, sleep disorder, cardiovascular problems, and stomach or digestion problems. Psychological complaints were feelings of unrest, of pressure, of helplessness, inner tension, and anxiety. In a Principal Component Analysis all nine complaints loaded on one component (Eigenvalue = 2.59). Accordingly, they were summed to yield one complaints score which was analyzed together with the answers to the other procrastination questions to yield the subset retained for the final diagnostic criteria.

#### 2.3.2. Aitken Procrastination Inventory (API)

Procrastination was assessed with the *Aitken Procrastination Inventory* [API; Aitken, 1982; German version by Helmke and Schrader (2000)]. It consists of 19 items with 5 response options (false, mostly false, sometimes false/sometimes true, mostly true, true). In the German version *sometimes false/sometimes true* had been replaced by *partly true*. Ten of the items are reverse coded. In an Exploratory Factor Analysis (EFA) with varimax rotation of the answers to a German translation of the API, Helmke and Schrader (2000) found three dimensions: *central trait procrastination* (factor 1), *lack of foresight* (factor 2), and *lack of punctuality* (factor 3). Similar factors were identified by Owens et al. (2008) in a principal axis factor analysis with an oblique rotation of the responses of American university students. They called the first factor *delayed task start*. The number of participants in these two studies was rather small for obtaining a stable three-dimensional solution (*N* = 117 and *N* = 152), therefore we relayed on a previous analysis performed with *n* = 939 students from our university (Patzelt and Opitz, 2005). In this analysis the structure of the answers to the 19 items had been established with a confirmatory factor analysis for categorical data (Mplus 3.1). Questionnaire content and response format were identical to the questionnaire presented to the current sample. Only the first factor covering the items No. 1 to No. 12 and No. 19 will be used in the following analyses. Cronbach’ss alpha established for these items was alpha = 0.91 for the respondents. The item loading highest was item No. 1, *I delay starting things until the last minute*. We called this factor *onset delay* following Svartdal et al. (2020). Table 1 presents the correlation of the sum of these items with the 13 procrastination items. For brevity, API in the following refers to this factor.

#### 2.3.3. Assessment of depression

The depression scale of the *Patient Health Questionnaire* (PHQ-9; Kroenke and Spitzer, 2002; Löwe et al., 2004) was used for assessment of depressive symptoms. It had been developed as a screening method to be applied by general practitioners. The depression scale includes 9 items for the symptoms of major depression according to the DSM-5 (American Psychiatric Association, 2013). It asks for the frequency of relevant symptoms in the past 2 weeks, using a 4-point rating scale (*not at all* to *almost every day*). Several studies confirm the reliability (Cronbach’s α = 0.88) and validity of the PHQ-9 (Löwe et al., 2004). The answers can be evaluated dimensionally as sum scores, or categorically following a diagnostic algorithm which differentiates major depression and other depressive syndromes. For a diagnosis of major depression at least five items including either the first or the second item had to be present on more than half the days in the past 2 weeks. Item No. 9 asks for suicidal tendencies and is counted already when answered *on some days*. The PHQ-9 allows also to grade depressive symptom severity. Scores ≥ 20 are assigned the severity rating s*evere*, scores from 15 to 19 *moderately severe*, and scores from 10 to 14 *moderate*.

### 2.4. Statistical analysis

Sequential analyses were conducted from the characteristic facets of procrastination found in the literature to the presentation of diagnostic criteria. The dimensionality of the newly presented procrastination items was explored first, followed by Best Subset Multiple Regression (BSMR) to find a subset of these items strongly related to the API. A latent class analysis (LCA) of these selected items identified several clusters. The groups defined by these clusters were compared with one another with respect to all procrastination items and several descriptive and clinically informative variables. The selected items were transformed to dichotomous criteria for pathological procrastination. The last step was to compare the selection of participants obtained with these criteria to the cluster solution.

The dimensionality of the procrastination items was explored with Exploratory Factor Analysis (EFA; SPSS Version No. 29) with varimax rotation. Item No. 10 was not included in the analysis as it does not inquire about procrastination behavior but its influence on relationships to other persons. Factorizing the items will clarify if the procrastination items do represent different aspects of procrastination behavior or vary only in item difficulty.

To select a subgroup of these procrastination items strongly related to the API, a BSMR was calculated (SigmaPlot 12.0). In this analysis the 12 retained procrastination items were searched for the optimal subset. The number of predictors is systematically increased and all possible predictor modes for a set of predictors are compared to find that model which explains most of the variance of the regressed variable (Brooks, 2016). This procedure successively increases the explained variance of the regressed variable, but less so with each additional inclusion of a predictor variable. Intercorrelations among the already selected variables and further variables will reduce the gain in explained variance with subsequent steps.

The selection procedure was continued until less than one percent would have been added to the variance explained by the already established best subset. For the final retained set of predictors the multiple regression information is presented. The results were examined for violation of requirements for this analysis. Correlations between the selected items and the API *onset delay* sum score were examined (Table 2). Homoscedasticity was ascertained by inspection of the plot of the predicted values of the API against the standardized residuals of the API. According to the value of 1.9 of the Dubin-Watson-Statistic the model is not impaired by auto-correlation and the variance influence factor VIF = 1.43 excludes multicollinearity. The final regression model was accepted.

**TABLE 2:** Summary of regression analysis for the six selected procrastination items predicting API onset delay scores.

Item	Criterion^a^	Best subset order^b^	Questions abbreviated	*B*	95% CI	*β*	*t*
			Constant	0.48	[−0.79, 1.76]		0.73
1	B	3	Interference with personal goals	1.32	[0.84, 1.71]	0.15	5.93
2	A	4	Delaying important tasks	2.09	[1.39, 2.80]	0.16	5.80
3	C1	1	Time spent procrastinating	2.63	[2.07, 3.20]	0.26	9.11
4	C2	2	Time pressure	2.44	[2.012, 2.77]	0.32	14.65
5	C3	6	Complaints	0.44	[0.26, 0.63]	0.10	4.70
6	C4	5	Below performance potential	1.26	[0.75, 1.77]	0.12	4.86

*n* = 990. The variables are arranged as in Table 1. All *t*-values for variables are significant (*p* < 0.001).

^a^Marker of the final diagnostic criteria.

^b^Sequence of extraction of variables in BSMR.

The selected procrastination items were analyzed to determine if they could differentiate between groups of procrastinators with varying degrees of severity and pervasiveness of their procrastination tendencies. LCA was used to identify distinct clusters of participants with different answer profiles. We utilized Latent Gold (Version 5.2; Vermunt, 2017) for this analysis. It offers a menu-driven model specification, graphical output, and standardized discrimination parameters. LCA determines clusters by assigning probands to clusters which minimize the intercorrelations of variables within these cluster. Comparison of different models is often based on the Bayesian Information Criterion (BIC). The number of clusters is systematically increased until a satisfactory solution is found. LCA is based on maximum likelihood estimation and suited to the analysis of metric, categorical, and ordinal data like the procrastination items. The assumptions, the steps to follow and the decisions possible to achieve a satisfactory latent class cluster solution are described in numerous publications (e. g. Schreiber, 2017; Nylund-Gibson and Ghoi, 2018; Weller et al., 2020). Porcu and Giambona (2017) provide a systematic introduction exemplified with an analysis of survey data with the program Latent Gold which we followed. An example of LCA applied to questionnaire data (Alcohol Use Disorders Identification Test; Babor et al., 2001) is presented by Rist et al. (2009).

The cluster variables and the remaining procrastination items were tested for differences between clusters with Kruskal–Wallis tests and Dunn–Bonferroni *post-hoc* comparisons. ANOVA and Dunn–Bonferroni tests were used for Age, Number of terms and the sum scores of API and PHQ-9. Overall *Chi*^2^ tests followed by *post hoc* pairwise *Chi*^2^ tests with Bonferroni correction were applied to the dichotomous items *sex*, *diagnose of depression*, and *study requirements not met*. A nominal type I error rate of alpha =0.001 was used for all analyses. In subsequent analyses the most severe cluster was split to compare participants with and without a diagnosis of depression. *Chi*^2^ tests, *t*-tests, and Mann–Whitney *U* tests were used for these comparisons. All statistical test reports are accompanied by effect size calculations performed as Cohen’ss *d* for metric variables, as *Eta*^2^ for ordinal variables and as Cramer’ss *V* for dichotomous variables. Significance level was set to *p* < 0.001 for all statistical tests.

## 3. Results

### 3.1. Dimensional structure of the procrastination items

The intercorrelations of the 12 items varied between *r* = 0.14 and *r* = 0.73. Three factors with Eigenvalues greater one ware extracted which explained 51% of the variance (37, 9, and 5% before and 19, 17, and 15% after varimax rotation). Factor 1 comprised items which assess procrastination behavior (items No. 3, 2, 7, 4) with factor loadings from 0.48 to 0.78 (time spent procrastinating). The items of factor 2 assess discomfort and unease related to procrastination (items No. 11, 5, 8, 13, 12) with factor loadings from 0.42 to 0.75 (discomfort or remorse). Factor 3 comprised items which address personal disadvantages of procrastination (items No. 1, 9, 6) with factor loadings from 0.66 to 0.72 (interference with personal goals). Intercorrelations of the factor scores were 0.07 ≤ *r* ≤ 0.17. The items derived from the various definitions capture three distinct facets of procrastination: behavior, affective impairment, and personal disadvantages.

### 3.2. Selection of procrastination items by BSMR

The BSMR procedure for the regression of the API scores onto the 12 diagnostic items started with the variable with the highest correlation and was stopped bevor the addition to the already explained variance fell below 1. For the finally retained set of predictor variables the multiple regression information is presented in Table 3. The API explained variance increased from *r*^2^ = 0.47 to *r*^2^ = 0.59, 63, 0.65, 0.66, and 0.67 in the successive variable selections, but the gain in explained variance fell below one at the next step. Total *R*^2^ of the model was *R*^2^ = 0.672 (*R^2^_*adj*_* = 0.669). The six collected items are displayed in Table 1.

**TABLE 3:** Model fit evaluation information for the latent class analysis of the six selected procrastination items.

Model	LL	BIC	AIC	Npar	L^2^	*df*	Class. Err.
1-cluster	−8562.08	17317.29	17180.15	28	4831.78	962	0.00
2-cluster	−7838.74	15918.89	15747.47	35	3385.10	955	0.05
3-cluster	−7691.21	1572.12	15466.41	42	3090.04	948	0.11
4-cluster	−7607.55	15553.09	15313.10	49	2922.73	941	0.14
5-cluster	−7560.41	15507.10	15232.82	56	2828.45	934	0.16
6-cluster	7521.62	15477.79	15169.23	63	2750.86	927	0.18
7-cluster	−7501.69	15486.21	15486.21	70	2711.00	920	0.19
Final model^a^							
6-cluster	−7488.34	15425.04	15106.68	65	2684.31	925	0.20

*n* = 990. LL, log-likelihood; BIC, Bayesian information criterion; AIC, Akaike information criterion; Npar, number of estimated parameters; L^2^, square of the likelihood; df, degrees of freedom; Class. Err., classification error.

^a^Four bivariate residuals are included as direct effects in the final 6-cluster model.

All predictors in the multiple regression (Table 2) contributed significantly (*p* ≤ 0.008).

According to the standardized regression coefficient *beta* = 0.32, No. 4, contributes most to the prediction of the API, followed by No. 3 *time spent procrastinating* (*beta* = 0.28). These two variables were chosen as the first ones in the BSMR. For the remaining items beta varied from *beta* = 0.10 to *beta* = 0.16. The BSMR did not select items evenly from the three different EFA domains. Only one item was chosen from the factor *discomfort or remorse*, two items from the factor *personal disadvantages* and three items from the factor *procrastination behavior*.

### 3.3. Latent class cluster analysis of the procrastination items

The items selected by the BSMR were analyzed as ordinal data in the LCA. A six-class model achieved the best fit according to both the lowest BIC and the lowest AIC for this solution (Table 3). Both indices decreased from the one cluster solution to the five-cluster solution to increase again for the succeeding model with seven classes. This model sorts the participants into six groups which differ in their answers to the procrastination items. The results for the models with one to seven clusters are presented in Table 3. Direct effects were set for two pairs of variables with particularly high, respectively low correlations which led to large bivariate residuals. These were (Table 1) No. 2, *delaying important tasks*, and No. 3, *time spent procrastination* (*r* = 0.73), No. 4, *time pressure*, and No. 6, *below performance potential* (*r* = 0.25). There remained one bivariate residual of 2.99 between No. 5 *complaints* and No. 4 *time pressure* (*r* = 0.23). The next three residual effects did not exceed 1.37 and the remaining nine residual effect were <1. The resulting classification error of 0.18 is acceptable considering the unequal cluster size (23 ≤ *n* ≤ 283).

**FIGURE 1:**
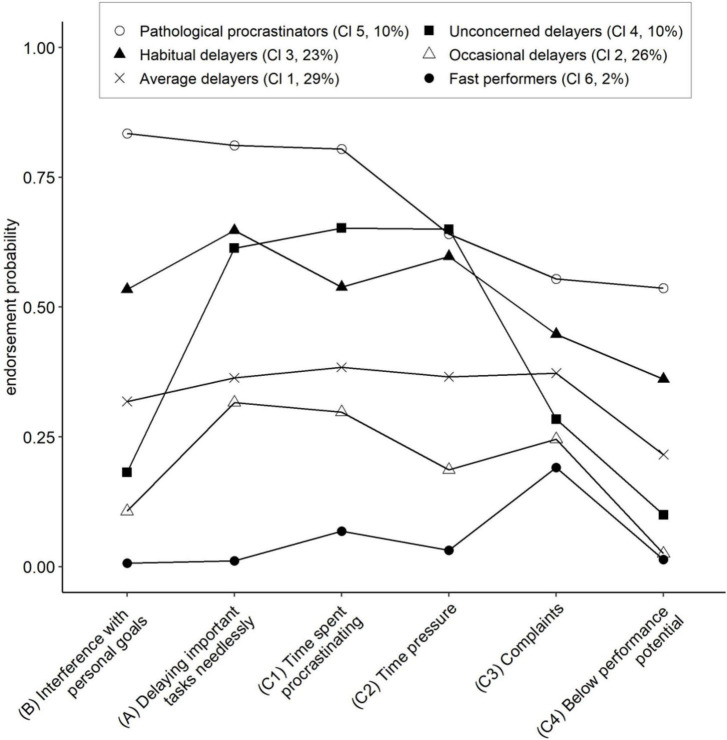
Profiles of the six clusters obtained with the latent class analysis for the six selected procrastination items. *n* = 990. Cluster names are followed by Cl 1–Cl 6 to indicate the order of the clusters. A, B, and C1–C4 designate the item position in the final diagnostic rule.

The endorsement probabilities for each of the six items in the five-class model are displayed in Figure 1. These and the class prevalence values allow to interpret the class profiles. The clusters are presented with their suggested names, the order of their extraction and the percentage of participants assigned to them. The variables on the x-axis were arranged to achieve a simple graphical impression, beginning with the variable which spreads the clusters most. The remaining variables are ordered according to their value for the upmost cluster, aiming at a downward slope of this curve. Table 4 displays the means of the six procrastination items for each cluster together with their API and PHQ-9 depression scores, Age, Gender, number of terms, and the number of students who have not met their study requirements. The clusters were also compared with respect to the seven procrastination items which have not been selected by the BSMR in Table 5.

**TABLE 4:** Cluster comparison for the six selected procrastination items, API, PHQ-9, and sociodemographic information.

Item	Procrastination items and descriptive variables	Statistics	Cluster 5 patho-logical procrasti-nators	Cluster 4 uncon-cerned delayers	Cluster 3 habitual delayers	Cluster 1 average delayers	Cluster 2 occasional delayers	Cluster 6 fast per-formers	All respondents	Dunn–Bonferroni^a^	Effect size^b^
	Participants	*n* (%)	101 (10)	100 (10)	230 (23)	283 (29)	253 (26)	23 (2)	990 (100)		
1	(B) Interference with personal goals	*M* (SD)	4.41 (0.53)	1.59 (0.51)	3.12 (0.64)	2.28 (0.64)	1.40 (0.50)	1.00 (0)	2.37 (1.11)	5 > 3 > 1 > 4 = 2	0.69
2	(A) Delaying important tasks needlessly	*M* (SD)	3.46 (0.58)	2.93 (0.62)	2.97 (0.95)	2.04 (0.31)	1.94 (0.33)	1.00 (0)	2.44 (0.75)	5 > 3 = 4 > 1 = 2	0.64
3	(C1) Time spent procrastinating	*M* (SD)	4.25 (0.61)	3.73 (0.69)	3.50 (0.70)	2.51 (0.55)	2.17 (0.39)	1.22 (0.42)	2.92 (0.95)	5 > 4 = 3 > 1 > 2	0.62
4	(C2) Time pressure	*M* (SD)	3.60 (1.22)	3.68 (1.15)	3.42 (1.09)	2.45 (0.90)	1.71 (0.70)	1.09 (0.28)	2.70 (2.70)	4 = 5 = 3 > 1 > 2	0.33
5	(C3) Complaints	*M* (SD)	5.06 (1.89)	2.20 (1.58)	4.05 (2.01)	3.41 (2.13)	1.52 (1.61)	2.01 (2.32)	3.24 (2.05)	5 > 3 > 1 > 4 > 2	0.22
6	(C4) Below performance level	*M* (SD)	3.21 (0.78)	1.40 (0.57)	2.39 (0.72)	1.89 (0.61)	1.05 (0.23)	1.04 (0.21)	1.86 (0.89)	5 > 3 > 1 > 4 > 2	0.60
	Diagnostic criteria met	*n* (%)	93 (93)	0	23 (10)	0	0	0	116 (100)		
	API	*M* (SD)	2.99 (0.46)	2.35 (0.49)	2.56 (0.47)	1.26 (0.44)	1.39 (0.53)	0.67 (0.28)	2.06 (0.74)	5 > 3 > 4 > 2 > 1	0.55
	PHQ-9 sum score	*M* (SD)	13.38 (5.36)	5.94 (3.98)	10.70 (5.23)	7.51 (4.47)	5.48 (3.94)	4.43 (3.79)	8.10 (5.28)	5 > 3 > 1 > 4 = 2	0.31
	Major depression	*n* (%)	44 (44)	2 (2)	50 (22)	24 (9)	8 (3)	1 (4)	129 (13)	5 > 3 > 1 = 2 = 4	0.37
	Gender (female)	*n* (%)	64 (63)	52 (52)	145 (63)	187 (66)	179 (71)	19 (83)	646 (65)	2 = 1 = 5 = 3 = 4	0.11
	Age (years)	*M* (SD)	25.72 (3.49)	23.3 (3.040)	24.19 (3.38)	23.42 (3.42)	23.39 (3.26)	23.52 (2.59)	23.82 (3.40)	5 > 3 = 1 = 4 = 2	0.24
	No. of terms	*M* (SD)	8.78 (5.25)	5.66 (4.59)	6.83 (4.97)	5.52 (4.15)	5.74 (4.05)	6.09 (4.71)	6.24 (4.60)	5 > 3 = 1 = 2 = 4	0.16
	Study requirements not met	*n* (%)	76 (76)	12 (12)	35 (22)	64 (23)	21 (8)	1 (5)	262 (27)	5 > 3 > 1 = 4 = 2	0.44

*n* = 990. Cluster 6 “fast performers” (*n* = 23) was not included in cluster comparisons. ANOVA was applied to age, PHQ-9, API and number of terms. Kruskal–Wallis tests were used for the procrastination items. Chi^2^ tests with Bonferroni correction were conducted for gender, study requirements not met, diagnostic criteria met and major depression. All other comparisons were Dunn–Bonferroni corrected.

^a^Dunn–Bonferroni *post hoc* comparisons “>” are significant (*p* < 0.001), “=” are not significant.

^b^Effect size was calculated as Cramer’s *V* for major depression, diagnostic criteria met, gender, and study requirements met. Effect size was calculated as *Eta*^2^ for all 13 procrastination items. Effect size was calculated as Cohen’s *d* for metric variables PHQ-D, API, Age and Number of Terms.

**TABLE 5:** Cluster comparison for the seven not selected procrastination items.

Item	Procrastination items	Statistics	Cluster 5 patholo-gical procrasti-nators	Cluster 4 uncon-cerned delayers	Cluster 3 habitual delayers	Cluster 1 average delayers	Cluster 2 occasional delayers	Cluster 6 fast performers	All respondents	Dunn–Bonferroni post hoc^a^	Effect size
	Participants	*n* (%)	101 (10)	100 (10)	230 (23)	283 (29)	253 (26)	23 (2)	990 (100)		
7	Preferring less important activities	*M* (SD)	3.46 (0.58)	2.93 (0.62)	2.97 (0.59)	2.04 (0.31)	1.94 (0.33)	1.00 (0)	2.44 (0.74)	5 > 4 = 3 > 1 > 2	0.35
8	Tasks not completed	*M* (SD)	3.40 (1.09)	1.43 (0.62)	2.23 (0.88)	1.66 (0.72)	1.66 (0.73)	1.04 (0.21)	1.82 (0.97)	5 > 3 > 1 = 4 = 2	0.16
9	Task aversion	*M* (SD)	3.02 (0.88)	2.28 (0.84)	2.65 (0.86)	2.17 (0.66)	1.96 (0.68)	1.39 (0.50)	2.31 (0.94)	5 > 3 > 4 = 1 > 2	0.40
10	Relationships affected	*M* (SD)	2.22 (0.87)	1.42 (0.55)	1.78 (0.89)	1.54 (0.62)	1.22 (0.44)	1.13 (0.34)	1.56 (0.69)	5 > 3 > 1 = 4 = 2	0.11
11	Discomfort or remorse	*M* (SD)	3.49 (0.76)	2.3 (0.92)	3.10 (0.87)	2.81 (0.85)	2.34 (0.85)	1.74 (0.86)	2.78 (0.94)	5 = 3 > 1 = 4 = 2	0.15
12	Suppressing thoughts	*M* (SD)	2.40 (0.97)	1.80 (0.80)	2.17 (0.88)	1.80 (0.80)	1.60 (0.70)	1.35 (0.71)	1.88 (0.86)	5 = 3 > 4 = 1 = 2	0.09
13	Keep thinking about tasks	*M* (SD)	3.46 (0.74)	3.11 (0.86)	2.31 (0.78)	3.08 (0.81)	2.92 (0.92)	2.26 (1.18)	3.11 (0.86)	5 = 3 > 1 = 4 = 2	0.04

*n* = 990. Cluster 6 “fast performers” (*n* = 23) was not included in cluster comparisons. Kruskal–Wallis tests with Dunn–Bonferroni correction were conducted for all variables.

^a^Dunn–Bonferroni *post hoc* comparisons marked “>” are significant (*p* < 0.001), “=” are not significant.

Several of the curves in Figure 1 have a rather similar profile and do not cross one another. The largest cluster (29%) is that of the *average delayers*, appearing in the middle with a rather horizontal curve for the first five variables and dropping at the last one, *below performance level*. This drop is shared by the cluster of *occasional delayers* (26%) below. This graph drops also markedly for the first variable *interference with personal goals*. The members of this cluster thus appear little threatened by procrastination related problems. This holds even more for the small group of *fast performers* (2%).

Mann–Whitney U-tests indicated significant differences between clusters for all six cluster variables (Table 4). Above the middle curve there are the clusters of *habitual delayers* (23%) and of *pathological procrastinators* (10%). The profile for the *habitual delayers* is marked by high values for *delaying important tasks needlessly*, *time spent procrastinating* and *time pressure*. *Interference with personal goals* appears less enhanced than these three variables. The upmost cluster of *pathological procrastinators* is marked by the highest values for five of the six variables with exception of variable No. 4, *time pressure*. Its values are about equal for *pathological procrastinators*, *habitual delayers*, and *unconcerned delayers*. *Time pressure* seems to be shared to an equal extent as soon as a certain critical intensity of procrastination tendencies is surpassed.

Strikingly different is cluster 4 of the *unconcerned delayers* (10%). Analogous to cluster 3, the *habitual delayers*, it reaches high values for *delaying important tasks needlessly*, *time spent procrastinating* and *time pressure*. But this cluster profile drops to low values for the remaining variables *interference with personal goals*, *complaints* and *below performance potential*. Thus, although procrastinating heavily, the members of this cluster claim not to be hindered in their strivings for achievement. The characteristics of this group are comparable with descriptions given for *active procrastinators* (Chu and Choi, 2005; Pychyl and Flett, 2012).

### 3.4. Differences between the clusters

Five of the six cluster variables differentiate the pathological cluster from the remaining clusters (Table 4). The exception is No. 4, *time pressure*, which is no less increased in *unconcerned delayers* and in *habitual delayers*. The pathological cluster differs significantly from the other clusters in API and PHQ-9 scores, but also in age, number of terms and study requirements not met. A significant difference is also found for the frequency of a diagnosis of depression derived from the PHQ-9. The pathological cluster is worse off both with respect to procrastination tendencies and by other behavioral and personal problems which accompany pervasive procrastination. Students had also been asked if they had looked for help for their procrastination problems and if so, they should check which kind of help on a list of possibilities. 35% of the pathological procrastinators and 22% of the habitual delayers, but less than nine percent from any of the remaining clusters had searched for help.

In addition, the cluster of *pathological procrastinators* differed from the other clusters in four of the seven not selected procrastination items (Table 5). No. 7, *preferring less important activities*, No. 9, *task aversion*, No. 8, *tasks not completed* and No. 10, *relationship affected* set the pathological cluster apart. However, these variables differentiate less between the remaining clusters. The last three items in Table 5, No. 11, *discomfort or remorse*, No. 12, *suppressing thoughts*, and No. 13, *keep thinking about tasks*, are similarly answered by the pathological procrastinators and the habitual delayers.

### 3.5. PHQ-9 depression results

The mean PHQ-9 scores of the six clusters and the frequencies of the diagnosis of Major Depression Syndrome are shown in Table 4. They correspond to the pattern of the PHQ-9 means across the six clusters. Forty-four percent of the *pathological procrastinators* and 22% of the *habitual delayers* received the diagnosis of Major Depression Syndrome. The frequency does not surpass 4% for the remaining three clusters.

To compare the results with those of Rozental et al. (2022), frequencies of graded depressive syndromes were calculated (Kroenke and Spitzer, 2002). Among *pathological procrastinators* 12% had severe depression and 32% moderately severe depression. Among *habitual delayers* 7% had severe depression and 15% moderately severe depression. Combining these two severity levels results in 44 and 22%, which correspond to the 52 and 28% frequencies of the Major Depression Syndrome obtained from the algorithm. The PHQ-9 sum score correlates *r* = 0.32 with *onset delay* across all participants.

The depression severity of the clusters varied in terms of the depression scores and the proportion of depressed students. Previous studies have reported correlations between depression scores and the API (Steel, 2007). In the pathological group, procrastination could be considered a consequence of manifest depression, given that 44% received a diagnosis of depression. To explore this further, we compared the procrastination tendencies of the depressed and of the non-depressed members of this cluster. Mann–Whitney *U* test were performed for all 13 procrastination items. Among the six items used for the cluster analysis, only the complaints score was significantly higher for the depressed members of this cluster, *U* (*N*_*D*_ = 44; *N*_*D*_ = 67) = 1825.500; *z* = = 3.97; *p* < 0.001). A diagnosis of depression did not enhance the response to the five procrastination items that directly concern procrastination behavior but was reflected in the number of bodily and psychological complaints.

Three items not selected by the BSMR (Table 5) differentiate between pathological procrastinators with and without a diagnosis of depression. Item No. 9, *task aversion*, Nr. 10, *relationship affected*, and No. 11, *discomfort or remorse* are significantly more endorsed by students with than without the diagnosis of depression, *U(N_*D*_* = 44; *N*_*D*_ = 67) = 1572.0; *z* = 2.55; *p* < 0.01. Pathological procrastinators with and without a diagnosis of depression do not differ in the behavioral aspects of procrastination, but depressed procrastinators experience even more emotional turmoil than do the other probands in that cluster.

### 3.6. Establishing the Procrastination Diagnostic Criteria (PDC-4/6)

To derive the final diagnostic criteria, the answers were combined into the categories of *present* and *absent* responses. As depicted in Table 1, the percentage of participants who met the criteria varied between 16 and 39% for the category of present responses. The complaints sum score was dichotomized at five symptoms, dividing the participants into 74% without and 26% with significant complaints. The questions on procrastination were reformulated as diagnostic criteria with different roles, as shown in Table 6. Two criteria, A and B, were designated as necessary for a diagnosis. Criterion A is derived from Item No. 2, *delaying important tasks*, and criterion B is derived from Item No. 1, *interference with personal goals*. Criterion A was chosen because it has the widest distribution across the six categories compared to the other criteria. Criterion B separates the *unconcerned delayers* from *pathological procrastinators* and from *habitual delayers*. Both criteria A and B must be met for a diagnosis of procrastination, along with at least two of the remaining four criteria.

**TABLE 6:** Diagnostic criteria for pathological procrastination (PDC 4/6) and their fulfillment frequency.

Diagnostic criteria		Met	
		***n***	**%**
During the last 6 months:			
(A) On at least half of the days, very important tasks were delayed past the adequate point in time, even though there was sufficient time to complete them.		386	39
(B) Procrastination interfered strongly with reaching personal goals.		158	16
(C) In addition, at least two of the following four criteria are met:		.	
(C1) More than half of the time available for completing important tasks was wasted procrastinating.		277	28
(C2) At least half of the plans to be completed were finished only under great time pressor or not at all, because of procrastination.		277	28
(C3) At least five bodily or psychological complaints have been experienced due to procrastination:		257	26
(a) Bodily complaints Muscle aches Sleep disorder Cardiovascular problems Stomach or digestion problems	(b) Psychological complaints Feeling of unrest Feeling of pressure Feeling of helplessness Inner tension Anxiety		
(C4) At least a quarter of the performance potential was impaired due to procrastination.		208	21
(D)^1^ The problems are not better explained by a different axis-I- or axis-II-disorder.			
A and B met		135	14
At least two out of C met		305	31
A and B and at least two out of C met		116	12

*n* = 990.

^1^Criterion D is to be judged person related by a clinical psychologist or a psychiatrist.

### 3.7. PDC-4/6 compared to the clusters

The PDC-4/6 identified 112 (12%) of the participants as diagnosed procrastinators (Table 4). Eighty percent of these were found in the *pathologi*cal cluster, and 20% were from the cluster of *habitual delayers*. Thus, only members of one additional cluster besides the *pathological cluster* were incorrectly identified as procrastinators by the criteria. Regarding these two clusters, 92% of the pathological cluster were correctly and 10% of the habitual delayers were incorrectly diagnosed. We compared these (*n* = 23) to the remaining members of this cluster (*n* = 207) on all six procrastination criteria. The incorrectly identified *habitual delayers* differed from the other members of this cluster only in meeting the B-criterion No. 1, *interference with personal goals* (Mann–Whitney *U* = 425.50; *z* = 7.44; *p* < 0.001). Importantly, none of the members of the cluster of *unconcerned delayers* was incorrectly diagnosed as pathological procrastinator, despite the similarity of their cluster profiles for three of the six cluster variables (Figure 1). Thus, the properties of the pathological cluster were retained to a large extent for the dichotomous PDC-4/6 criteria.

### 3.8. PDC-4/6 compared to selection by API

The API scores were dichotomized at 3.0 to separate heavily procrastinating persons (*n* = 112; 11%) from less procrastinating persons (*n* = 878; 89%). The value of 3.0 was chosen to yield participants with scores indicating high levels of procrastination which matched the number of PDC-4/6 - diagnosed pathological procrastinators (*n* = 116; 10%). Of those probands 47% were also marked by the API, but 53% were not identified by the API. Of those not diagnosed as pathological procrastinators, 7% were identified by the API as heavy procrastinators.

Comparing the API dichotomous frequencies to the clusters, 49% of the positive scores were assigned to the pathological cluster, 44% to the habitual delayers, 5% to the unconcerned delayers and two percent fell to the average delayers. Thus, an API cut score high enough to select about the same number of participants as the number of participants in the pathological cluster is still worse in identifying members of the group of pathological procrastinators than are the PDC-4/6.

## 4. Discussion

### 4.1. Definitions organized along three dimensions

The investigation began by scrutinizing definitions of procrastination naming essential facets to be considered when characterizing procrastination. Obviously, procrastination is a complex volitional and behavioral problem with various cognitive and emotional accompanying features. They were retained in 13 questions with a Likert style answer format. According to the EFA, they correspond to *procrastination behavior*, *accompanying affective impairment* and *resulting personal disadvantages*. The BSMR selected only one item from the affective impairment factor, with the remaining five items being assigned to the other two factors. Procrastination behavior and the resulting personal disadvantages directly reflect the problems associated with procrastination, while the affective factor items capture the accompanying emotional burden.

### 4.2. Onset delay assessed with the API

In their content analysis of current procrastination questionnaire items Svartdal et al. (2020) differentiate between *onset delay*, *sustained goal pursuit delay* and *delay in reaching the intended goal*, with *onset delay* to characterize strong procrastinators. According to their analysis, seven items of the API focus on delay of onset of goal directed activities. All seven items were assigned with high loadings to the first factor in the CFA by Patzelt and Opitz (2005), which we relied on in our analyses. Following Svartdal et al. (2020), this factor was named *onset delay*. The content of the selected six items seems suitable to reflect *past negative consequences*, which Svartdal and Nemtcan (2022) found crucial for separating maladaptive and irrational delay from other forms. The six items chosen because of their correlation with the API thus seem suitable to derive criteria for identifying maladaptive and irrational delay.

### 4.3. The subgroups found with the latent class clustering procedure

The intercorrelations of the procrastination items were mostly in the range of 0.20 ≤ *r* ≤ 0.50 and thus considerably lower than those of items from a unidimensional questionnaire. In addition to the pathological procrastinators, we identified five additional clusters. These results resemble the results of a former LCA conducted with the eight procrastination items of the initial PDC-5/8 (Rist et al., 2019). However, in this analysis more probands were sorted into the pathological class (13%) than in the now reported analysis (10%).

The clusters are not defined by their scores in questionnaires, but by their answers to single items. Nonetheless, they correspond to clusters identified by conventional questionnaires assessing procrastination, personality traits and depression, as employed by Rozental et al. (2015). Their analyses were conducted on a sample of students who had responded to the offer of treatment for procrastination. Consequently, their cluster of *severe procrastinators* was larger (22%) than our cluster of *pathological procrastinators*. They found three more clusters of *average*, *well-adjusted* and *mild procrastinators*, resembling our clusters of *habitual*, *average*, and *occasional delayers*. They also found a cluster of *primarily depressed* students, which we did not find. The offer of treatment may have selected more students who suffered from their procrastination than we reached in the random poll. Furthermore, Rozental et al. (2015) did not identify clusters of *unconcerned delayers* or *fast performers* whom we would not expect among students seeking advice or treatment. Grunschel et al. (2013) differentiated types of academic delayers in a profile analysis and also found a cluster of *successful pressure-seeking delayers*, resembling our *unconcerned delayers*.

### 4.4. Characterizing the clusters

Pathological procrastinators are clearly separated from the other clusters. The largest cluster, CL 1 (29%), consists of *average delayers*. Their profile is located between Cl. 3 of *habitual delayers* and Cl 2 of *occasional delayers*. Their depression scores follow this pattern. This cluster resembles one of the clusters identified by Rozental et al. (2015) and called “*well-adjusted procrastinators*.” In their study this was also the largest cluster (28%). Based on an extensive meta-analysis van Eerde (2003) characterized the typical procrastinator as having only average problems from procrastination and being only moderately affected in mood. This seems to characterize also the second largest cluster 2 of *occasional delayers* (26%). Their profile differed from that of the *average delayers* predominately by the lack of *interference with personal goals*, followed by less *time pressure*. Their depression scores are the lowest, close to those of the *fast performers*. Thus, although this group practices procrastination, it is not hampered by it.

Like Rozental et al. (2015) we found two clusters more afflicted by procrastination than the average delayers. Cl 3 (23%) of the *habitual delayers* reached higher values than the *average delayers* on all six variables. Also, their depression score were higher than those of the *average delayers*. CL 5 (10%) of the *pathological procrastinators* differs from CL 3 by even more extensive procrastination. However, this cluster is not significantly different from CL 4 of the *unconcerned delayers* regarding their answers to *time pressure*. Cl 5 obtained the highest depression score and the most diagnoses of major depression (44%) of all clusters. Of the *habitual delayers* of Cl 3, 22% obtained a diagnosis of major depression. The proportion of participants with major depression does not surpass 9% in the remaining four clusters.

The *unconcerned delayers* of CL 4 are the only group with a cluster profile that crosses other profiles (Cl 1 and Cl 3) and even reaches Cl 5 of the pathological procrastinators in *time pressure*. Their depression scores are even lower than those of the *habitual delayers*. This pattern of negating interference with personal goals and the absence of impaired mood corresponds to the characteristics of active procrastination (Chu and Choi, 2005; Chowdhury and Pychyl, 2018). As Chowdhury and Pychyl (2018) point out, this construct does not capture a homogeneous group. The unconcerned delayers have the second largest value for the item *time spent procrastinating*. That speaks for a felt waste of time and against the appearance of completely undemanding, possibly intentional procrastination. The depression scores of this group are not the lowest ones but surpass those of the occasional delayers and are not significantly different from those of the average delayers. With respect to the complete cluster solution and the derived dichotomous criteria, the appearance of this cluster supports its validity. None of the students diagnosed as procrastinators by the dichotomous criteria fell into this group as they did not fulfill the B criterion (see Table 6). However, the dichotomous criteria did not precisely capture the complete cluster of *pathological procrastinators* but ignored a small number of these and instead identified erroneously participants from CL 3 of the *habitual procrastinators* as pathological procrastinators. But this lack of precision should not hinder from the application of the diagnostic criteria.

### 4.5. Depression and procrastination

The prevalence of Major Depression Syndrome was found to be 44%, indicating a considerable risk for depression symptoms in this group. The mean depression score and the frequency of the diagnosis of Major Depression Syndrome were considerably higher in this sample than in another German student sample by Bailer et al. (2008). These authors reported 6% of students diagnosed with major depression and 8% with other depressive syndromes. In contrast, our sample had a frequency of 18% for Major Depression Syndrome, making it three times more common. However, Rozental et al. (2022) found even higher mean scores for depression (*M* = 8.95) in their sample than we did (*M* = 8.10). The high levels of depression in both studies may be due to selection procedures. Rozental et al. (2022) recruited participants through an online survey for procrastination and personality features, with access to a lecture on procrastination as reward. Our study recruited participants randomly through a survey, with a response rate of 10%. Participants in both studies may have been more likely to participate if they recognized own procrastination tendencies.

In our sample, depression scores and frequency of Major Depression Syndrome diagnosis were correlated with the severity of procrastination clusters. However, the diagnosis of Major Depression Syndrome only influenced answers to the *complaints* item and not to the other five items. Therefore, depression accompanies procrastination but only partially influences the presentation of procrastination symptoms.

### 4.6. Dichotomous criteria item selection

BSMR selected six out of 13 procrastination items according to their correlation with the API. The first selected variable (item Nr. 3; *time spent procrastinating*) explained 47% of the variance. The variables selected in subsequent steps added less explained variance, with only about 1% contribution in the end. The decision to terminate subset selection was arbitrary, intending to capture diverse procrastination facets while excluding variables that added less than 1% of explained variance.

### 4.7. Using the criteria

Dichotomous criteria were created by combining the response options for the two categories *present* and *absent*. For a diagnosis of pathological procrastination two specific dichotomous criteria must be met together with two from the remaining four optional criteria. One may be tempted to use the criteria directly, skipping the original procrastination questionnaire (Appendix, PDC-4/6). The wording of the criteria follows these questions, but they are formulated as statements, not as questions. It would not be wise to simply use the dichotomous criteria reformulated as questions to spare the presentation and evaluation of the original items. The decision if one fulfills a criterion or not should be based on the original questions by considering the critical response options.

Instead of the eight criteria that were previously used, only six procrastination items (PDC-4/6; see Appendix) are now used for criteria. For additional psychopathological information we recommend the presentation also of the remaining procrastination items (Table 1). The information gathered with these additional items (see PDCQ-12, Appendix) characterizes the life situation of heavily procrastinating persons. The example of item No. 9 on task aversiveness, *did the tasks you postponed cause reluctance or aversion?* (Table 1) may illustrate how valuable information can be gained through such a more extensive record of core features of procrastination: The aversion to tasks is stronger in procrastinators than in non-procrastinators and is an important condition for the pronounced avoidance seen in many afflicted persons. For the clinical diagnostic assignment, however, the item is not sufficiently selective. Even people with less procrastination tendencies experience reluctance and resistance to unpleasant tasks at least occasionally. The crucial difference lies in the ability to self-regulate despite aversion and this must be focused on accordingly in the intervention. Thus, in a future version of the questionnaire, item 9 should be revised, e. g. to *in case of unpleasant tasks I delay for a particularly long time*.

Within our actual solution, researchers or clinical and educational psychologists have the option of using only the six questions related to the criteria for the diagnostic assignment as pathological or non-pathological procrastination (PDCQ-6 in the Appendix) or to use all 12 questions (PDCQ-12 in the Appendix both for a diagnosis and for obtaining comprehensive information about the individual symptom characteristics in addition.

When using the PDCQ-6 or PDCQ-12 for clinical diagnosis one should also consider comorbidity in an additional differential diagnostic analysis. To assess the risk of accompanying depressive symptoms the PHQ-9 is a first psychometric choice which was also used by Rozental et al. (2022) to differentiate grades of depression. Attention-Deficit-Hyperactivity Disorder (ADHD) is a second disorder with enhanced prevalence of procrastination. In a study with 456 students ADHD was present in 21% of the procrastinating students and about four times more frequent than for non-procrastinating students (Rist et al., 2011). Vice versa the connection we found between both disorders is even more obvious: The prevalence of procrastination among ADHD-sufferers was about 50% compared to 12% for the participants without ADHD. This group is at a high risk to develop severe behavioral postponement. Inattention is the dominant symptom in ADHD which is correlated with general procrastination (Niermann and Scheres, 2014). More specifically, prospective memory mediates at least partially a link between ADHD symptoms and procrastination (Altgassen et al., 2019). Procrastination may be tied to a history of ADHD and screening for ADHD symptoms should accompany the procrastination diagnostics as recommended for symptoms of depression.

The development of the diagnostic criteria focused on pathological procrastinators in a clinical sense, but the items on which the criteria are based may as well be used to identify a more broadly defined group of delayers. A less restrictive rule might combine the groups of pathological procrastinators and habitual delayers and separate this new group from the remaining clusters, particularly from the unconcerned delayers. This should be useful for answering questions related to pervasive delay in general, but clearly distinguished from unconcerned delay.

### 4.8. Limitations

The invitation to participate in the investigation explained that the purpose of the study was to learn about procrastination and its consequences for students. This perspective did only attract a small proportion of those addressed and may have motivated more students who were familiar with procrastination to take part than those who were less hindered by it. This might explain the rather high percentage of students who fulfilled the criteria for *pathological procrastination* and the small number of *fast performers*. Evidence for such a selection was provided by the PHQ-9 scores of the participants. Their mean PHQ-9 score was more than three times as high as the mean score of a comparative German student sample that was not approached with a focus on procrastination (Bailer et al., 2008). The reproducibility and stability of the clusters should be explored in different samples, possibly with a different composition of more and less procrastinating individuals. The steps followed here, from BSMR via LCA to dichotomous criteria, should be repeated to test for fluctuations in the selection of items and deviations of dichotomous criteria from the now identified clusters. Acceptance of the suggested dichotomous criteria requires evidence of their stability. A crucial step was selecting a set of procrastination items according to their relation to the factor *onset delay* of the API. A different number of predictors could have been retained, but as the variables extracted first explain considerably more variance than later ones this might not have substantially changed the prediction of the API values. The LCA performed with these retained items sorted the probands into clusters according to the pervasiveness of procrastination symptoms. The emergence of the pathological cluster guided the formation of the diagnostic criteria. The final step to arrive at the dichotomous criteria was concatenating the response options into two categories, *present* and *absent*. Data from procrastination clients from our special outpatient clinic for procrastination helped to decide which response options to concatenate. We aimed to capture about 30% of the participants in the *present* category of each item to achieve a limited total number of diagnosed procrastinators. This could have been done differently, such as by accepting only the most difficult answer choice as *present* and combining all other choices into the *absent* category. However, this would have resulted in the diagnostic criteria only identifying a part of the pathological cluster.

Discussion of matters of diagnosis and classification has accompanied psychiatry and clinical psychology for many years. Psychiatric classification serves the purposes of enhancing communication, determining relations to other conditions and providing additional information within a classification system (Lilienfeld et al., 2016). The suggestion of diagnostic criteria for procrastination should be subjected to the same considerations as modifying already existing diagnostic criteria, as outlined by Doust et al. (2017). Their checklist demands that such a definition is repeatable and reproducible to improve the consistence of clinical decision making. The number of people affected is also important in understanding benefits, harms and resources needed. According to their check list, the “most important feature of a disease definition is its ability to accurately predict clinically meaningful outcomes” (p. 1022). Despite the limitations and the need for further consolidation of validity and reliability, the suggested diagnostic criteria offer the first steps toward a viable alternative to relying only on dimensional assessment of clinically relevant procrastination.

## Data availability statement

The raw data supporting the conclusions of this article will be made available by the authors, without undue reservation.

## Ethics statement

Ethical review and approval was not required for the study on human participants in accordance with the local legislation and institutional requirements. The patients/participants provided their written informed consent to participate in this study.

## Author contributions

E-MF and JW-L prepared and conducted the data collection for their diploma thesis under the surveyance of FR and ME. AH and FR conducted the analysis of the data set. FR and ME prepared the manuscript. All authors contributed to the article and approved the submitted version.

## Footnotes

1
https://www.uni-muenster.de/Prokrastinationsambulanz/

2
https://www.unipark.com/en/

## Appendix

### Initial Proposal of Procrastination Diagnostic Criteria (PDC-5/8^1^) (Höcker et al., 2017):

In the last 6 months

A. On at least half of the days very important tasks were delayed past the adequate point in time even though there was enough time available.

B. Procrastination interfered strongly or very strongly with reaching personal goals.

C. In addition at least three of the following six criteria are fulfilled:

C1) More than 50% of the time available for completing the task was spent procrastinating.
C2) On at least half of the days other, less important tasks were preferred, even though one wanted to start working on the important task.
C3) The delayed tasks caused at least half of the days aversion and animosity.
C4) At least half of the plans to be completed within the last half year were finished only under great pressure of time or not at all because of procrastination.
C5) Due to procrastination at least 50% of the achievement potential is impaired?
C6) There are at least five bodily or psychological complaints due to procrastination.

D. The problems are not better explained by another physical or mental disorder.

For the diagnosis “Pathological procrastination” Criterium A and B must be fulfilled, additionally three of the following C-Criteria in accordance with the Questionnaire PDCQ-8.

^1^ *Criterium D is not counted in our PDC-8 or PDC-6 naming scheme and evaluation rule. It has to be judged person-related by a clinical psychologist or psychiatrist.*

### Procrastination diagnostic criteria questionnaires: PDCQ-6 and PDCQ-12

This inventory assesses the habit of postponing personally important activities which one intended to do (procrastination) and its effect on your personal life. Please read these questions attentively and decide for each question which of the answers best describes your situation.

All questions concern the time span of the last 6 months.

**1 (A) Have you delayed very important tasks past the adequate point in time, despite sufficient time for it had been available?**

• not at all
• on some days
• on more than half the days
• almost on every day

**2 (B) To which extent did procrastination hinder you from reaching your personal goals?**

• not at all
• little
• medium
• strongly
• very strongly

**3 (C1) Remember important tasks you procrastinated: How much of the time available for the tasks did you spend procrastinating?**

• no time spent procrastinating
• up to 25% of the time spent procrastinating
• up to 50% of the time spent procrastinating
• up to 75% of the time spent procrastinating
• more than 75% of the time spent procrastinating

**4 (C2) Think of important plans which you wanted to finish during the past half year. How many of these plans did you finish only under great time pressure (or not at all), because you procrastinated?**

• none finished under time pressure (or not at all)
• up to 25% finished under time pressure or not at all)
• up to 50% finished under time pressure (or not at all)
• up to 75% finished under time pressure (or not at all)
• more than 75% finished under time pressure (or not at all)

**5 (C3) Has procrastination led to any of these complaints?** Please mark all your complaints:

**Bodily complaints:**

• muscle aches
• sleep disorder
• cardiovascular problems
• stomach or digestion problems

**Psychological complaints?**

• feeling of unrest
• feeling of pressure
• feeling of helplessness
• inner tension
• anxiety

**6 (C4) Starting from a performance potential of 100%: To which extent (%) did you remain below your performance potential because of procrastination?**

• no loss
• up to 25% loss
• up to 50% loss
• up to 75% loss
• more than 75% loss

**7 Did you choose other, less important activities despite you wanted to start with your more important task?**

• not at all
• on some days
• on more than half the days
• almost every day

**8 Did the tasks you postponed cause reluctance or aversion?**

• not at all
• on some days
• on more than half of the days
• on almost every day

**9 Has procrastination affected your personally important relationships to significant others?**

• not at all
• on some days
• on more than half of the days
• on almost every day

**10 Did procrastination cause strong discomfort or remorse?**

• not at all
• on some days
• on more than half of the days
• on almost every day

**11 Do you keep thinking about the postponed task while procrastinating?**

• not at all
• on some days
• on more than half of the days
• on almost every day

**12 Do you try to suppress thoughts about the postponed task?**

• not at all
• on some days
• on more than half of the days
• on almost every day

_______________________________________________________

***Evaluation for PDC-4/6: Pathological Procrastination present or absent?**

Features of Items 1–4 are present, if the answer meets the last two response options.

C3 (Item 5) requires at least 5 complaints.

C4 is present if the answer to Item 6 meets one of the last three response options.

**For a diagnosis of pathological procrastination:**

• The A- and B-criteria together (Item 1 and 2) are met
• and two out of the remaining four C-criteria (Items 3–6) are met.
